# Solitary Rectal Ulcer Syndrome Mimicking Perianal Crohn's Disease

**DOI:** 10.7759/cureus.23733

**Published:** 2022-04-01

**Authors:** Charleston R Powell, Jessica A Lawson, Phillip C Lindholm

**Affiliations:** 1 Internal Medicine, Weed Army Community Hospital, Fort Irwin, USA; 2 Pathology, Tripler Army Medical Center, Honolulu, USA; 3 Gastroenterology, Madigan Army Medical Center, Tacoma, USA

**Keywords:** crohn's disease (cd), gi endoscopy, rectal ulcer, fistula, perianal disease, gastroenterology, inflammatory bowel disease, solitary rectal ulcer syndrome

## Abstract

Solitary rectal ulcer syndrome (SRUS) is an uncommon condition that presents with non-specific symptoms shared by other disease processes. This case report shares a unique presentation in which a patient was thought to have fistulous perianal Crohn's disease then underwent treatment with infliximab but was ultimately found to have SRUS. The prognosis and treatment of SRUS vary greatly from inflammatory bowel disease. Making the correct diagnosis is imperative when considering Crohn's disease and its mimickers.

## Introduction

Solitary rectal ulcer syndrome (SRUS) has an incidence of one out of 100,000 persons per year. The precise pathogenesis has not been identified but is thought to be related to local ischemia leading to ulceration. Presenting symptoms vary and include tenesmus, rectal prolapse, constipation, and bleeding. Up to 26% of patients have no symptoms before diagnosis [1]. SRUS can afflict younger adults yet is typically seen in the third to fourth decade of life [2]. Rectal prolapse, intussusception, paradoxical contraction of pelvic floor muscles, and trauma can all precede a diagnosis of SRUS. There are many treatment options, yet full remission of disease is difficult to obtain. 

The name SRUS is a misnomer, as it can also present with polypoid lesions or non-ulcerated erythema. Ulcers are only found in 40% of patients. Of these, only 20% have solitary ulcers [2]. Because of the overlap in symptoms and endoscopic similarities between SRUS and other diseases, a careful review of history, endoscopic findings, and histology is required for diagnosis. We present a case of SRUS with a perianal fistula that was initially concerning for perianal Crohn's disease.

This article was previously presented as an e-poster at the ACG 2020 Virtual Annual Scientific Meeting & Postgraduate Course on October 23-28, 2020.

## Case presentation

**Figure 1:**
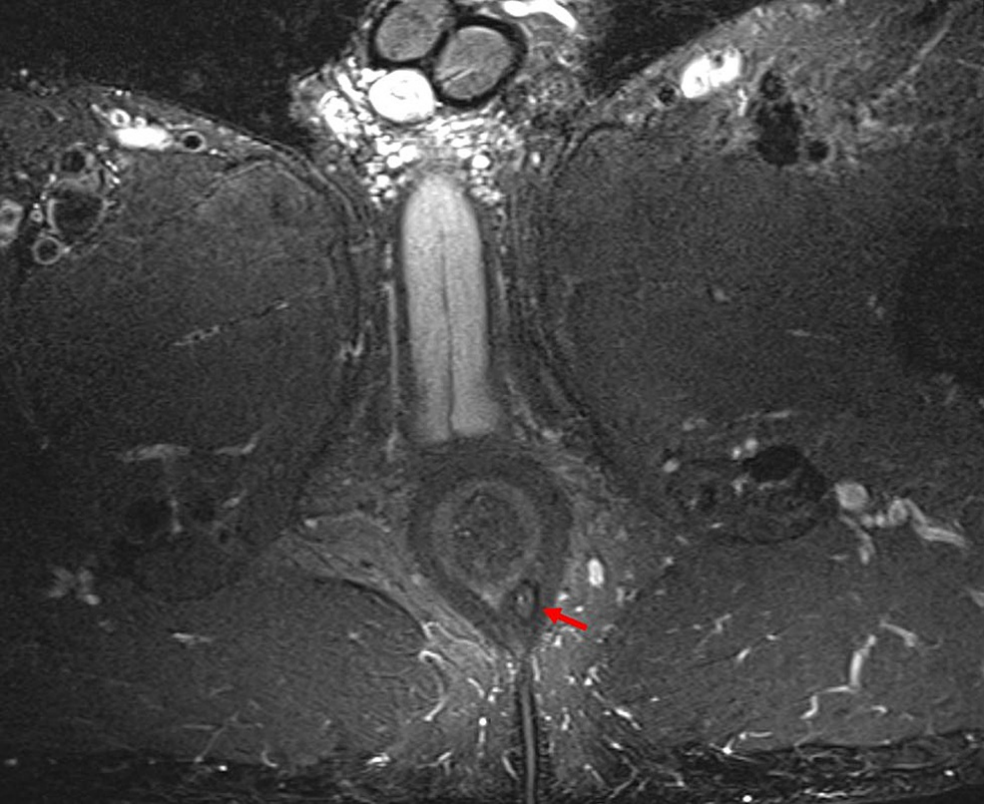
MRI of perianal fistula Axial, T2-weighted MRI image showing fistulous tract adjacent to the left posterior aspect of the rectum (arrow).

A 33-year-old male special-operations soldier experienced 10 years of tenesmus, crampy abdominal pain, constipation, and intermittent hematochezia. Throughout this period, he had several presentations to primary care providers where he was prescribed stool softeners for constipation and topical corticosteroids for self-reported hemorrhoids. He had never undergone a formal evaluation of his hematochezia but was ultimately referred to a gastroenterologist at a separate institution when he had positive fecal occult blood tests that were ordered routinely for a career-focused health assessment. He underwent colonoscopy, including a terminal ileum examination that showed ulcerated, erythematous, and granular mucosa of the rectum as well as a potential perianal fistula. Pathology of the rectal biopsy showed scattered neutrophils in the lamina propria invading the glandular epithelium with a differential diagnosis of inflammatory bowel disease, infectious, ischemic, or acute self-limited colitis. An MRI was ordered to follow up on the potential anal fistula and confirmed the presence of a 2.9 cm intersphincteric fistula at a 5 o'clock position (Figure 1). Laboratory results were notable for normal c-reactive protein levels as well as white blood cell count. CT enterography was ordered to evaluate small bowel, and it did not show evidence of small bowel involvement. It was then decided that the most likely etiology was Crohn's disease, and the patient was initiated on infliximab 5 mg/kg. Before arriving at our institution, he planned to continue infliximab and have an interval assessment of his proctitis with endoscopy and his fistula with repeat imaging.

**Figure 2:**
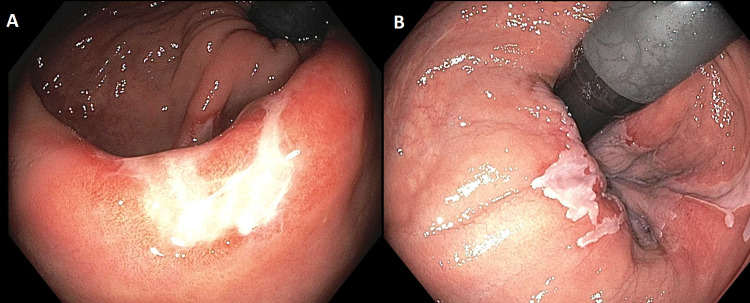
Follow-up colonoscopy after six months of infliximab Endoscopic views of prominent rectal ulcer with patchy, white exudate.

**Figure 3:**
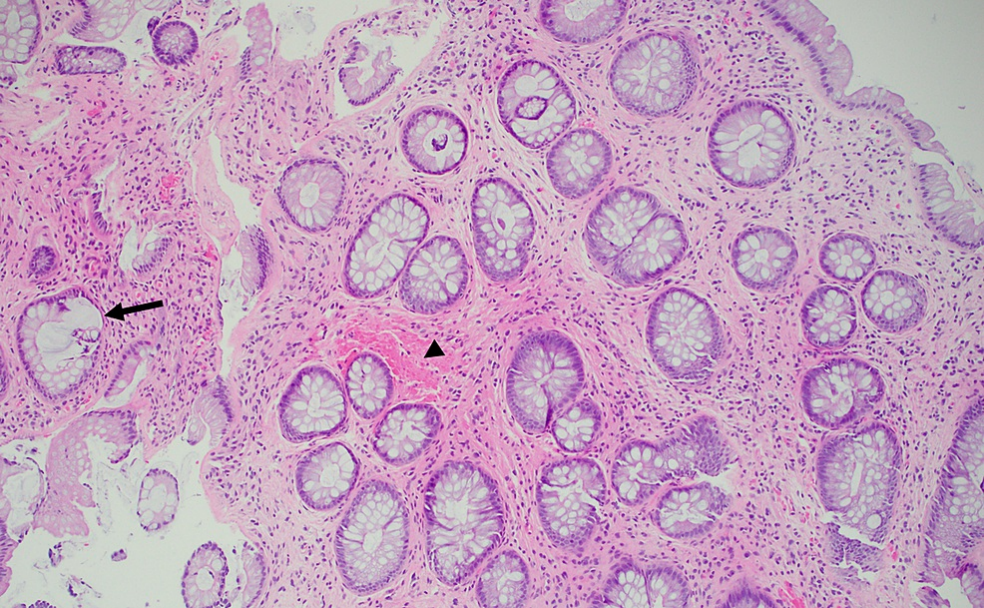
Pathology of rectal ulcer biopsy Histology showing crypt distortion (arrow) and smooth muscle proliferation (arrowhead).

Upon arrival at our institution, the patient reported modest improvement in his abdominal pain but the persistence of tenesmus and hematochezia. A follow-up colonoscopy after six months of infliximab showed a prominent rectal ulcer with patchy exudate (Figure 2). Serum trough infliximab levels were therapeutic at 19 mcg/mL with no antibodies. Biopsy demonstrated hypertrophic, disorganized muscularis mucosae with smooth muscle and collagen extending into the lamina propria with crypt distortion that is typical of SRUS (Figure 3). CMV immunostaining was negative.

His persistent symptoms despite immunotherapy and the histologic appearance of his rectal ulcer argued against inflammatory bowel disease as etiology for his isolated anorectal disease. Infliximab was stopped, and the patient was referred to a pelvic floor physical therapist after anorectal manometry confirmed dysnergic defecation with the paradoxical response of anal sphincter and pelvic floor with simulated defecation. Flexible sigmoidoscopy performed after three months of pelvic floor physical therapy revealed improved ulceration and no evidence of fistulae.

## Discussion

This case illustrates the difficulty in achieving a diagnosis of SRUS, especially when presenting with a perianal fistula. Anorectal fistulae can be the consequence of inflammatory bowel disease, malignancy, chronic constipation, and trauma. Fistulae in the setting of SRUS are rarely described, and our literature search came across only one such case where a patient with severe SRUS developed a rectovaginal fistula [3]. Fistula in the setting of chronic constipation, however, is more common. The fact that the initial biopsy showed active colitis paired with the finding of a fistula is likely a contributing factor to why he was originally classified as having inflammatory bowel disease.

Diagnosis of SRUS begins with endoscopy and typically shows abnormal mucosa of the anterior rectal wall. The endoscopic findings can be diverse in both appearance and quantity, ranging from ulcerated to nodular and solitary to multiple ulcers [4]. Biopsy with careful histopathologic review and a high index of suspicion is necessary. Microscopically, hypertrophy of smooth muscle fibers from the muscularis mucosa that extend into the lamina propria is highly sensitive to SRUS [2]. Our patient’s biopsy demonstrated this classic pattern as well as notable crypt distortion, which is also seen in Crohn's disease and, more commonly, ulcerative colitis[5]. The lack of granulomatous inflammation or lymphoid aggregates that are seen more commonly with Crohn's disease [6] and clinical history of constipation was not consistent with colonic Crohn's disease.

An estimated 26% of patients receive an incorrect diagnosis before confirmation of SRUS [7]. Given the spectrum of endoscopic findings possible, inflammatory bowel disease or neoplasm are generally considered. An incorrect diagnosis can expose patients to therapy with steroids and immunotherapy. This can lead to serious side effects and, in this case, important career ramifications. SRUS is a functional disorder of defecation. There are no robust guidelines for medical treatment of SRUS nor significant studies comparing strategies, but data suggests that patients can have improvement of their disease course with well-tolerated therapies such as sucralfate, high-fiber diets, and biofeedback. Ulceration persists in 43% of patients, and many patients will suffer from anorectal dysfunction despite aggressive therapy [8]. Our patient underwent years of pelvic floor physical therapy and required regular clinical follow-up to reiterate proper bowel habits and the use of polyethylene glycol. His course included ongoing bleeding, constipation, and tenesmus, yet he was allowed to maintain his career.

## Conclusions

SRUS is an uncommon disease that presents with a variety of symptoms that are seen more commonly in other conditions. It is a benign functional disorder of defecation that can improve without aggressive surgical or medical treatment. Missing this diagnosis can expose patients to intensive medical therapies and procedures, cause psychological distress and limit one's career options. We strongly suggest considering SRUS as an inflammatory bowel disease mimicker for isolated rectal disease even when fistulae are present. This can be accomplished through challenging alternative diagnoses when a patient does not improve as expected when on appropriate therapy for their original diagnosis. 
